# Moyamoya disease: epidemiology, clinical features, pathogenesis, diagnosis and therapeutic interventions

**DOI:** 10.1186/s43556-025-00318-y

**Published:** 2025-10-10

**Authors:** Xinyue Cheng, Ying Cao, Junbo Duan, Min Zhou, Shoudong Ye, Yuqing Zhu

**Affiliations:** 1 Center for Stem Cell and Translational Medicine, School of Life Science, Anhui University, Hefei, 230601 China; 2 Department of Critical Care Medicine, The First Affiliated Hospital of USTC, Hefei, 230001 China; 3 Division of Life Sciences and Medicine, University of Science and Technology of China, Hefei, 230026 China

**Keywords:** Moyamoya disease (MMD), RNF213, IPSC-based cell therapy, Gene editing

## Abstract

Moyamoya disease (MMD) is a rare cerebrovascular disorder characterized by progressive stenosis of the intracranial internal carotid arteries and the development of compensatory, fragile collateral vascular networks at the skull. Emerging evidence suggests that the pathogenesis of MMD involves genetic/epigenetic predisposition, dysregulated immune responses, and environmental triggers. Notably, the RNF213 p.R4810K variant has been identified as a key genetic susceptibility factor, particularly in East Asian populations. However, the molecular mechanisms underlying disease progression remain incompletely elucidated, primarily due to the limited availability of patient-derived cerebrovascular tissues and the lack of animal models that faithfully recapitulate the full spectrum of human MMD pathology. These constraints have impeded the development of targeted therapeutic interventions. Diagnostically, digital subtraction angiography (DSA) continues to serve as the gold standard for diagnosing MMD, enabling detailed visualization of steno-occlusive lesions and characteristic moyamoya vessels. Current clinical management relies predominantly on surgical revascularization to enhance cerebral perfusion, yet this strategy does not alter the fundamental disease process. Recent advances in patient-derived vascular organoids and serum-stimulated cellular models have facilitated drug screening and biomarker identification. In this review, we provide a systematic overview of the epidemiology, clinical manifestations, and genetic landscape of MMD, with a focus on recent progress in deciphering its molecular basis. We further discuss the transformative potential of induced pluripotent stem cell (iPSC) technology, particularly when combined with CRISPR-based gene editing, for modeling MMD vasculopathy, investigating the functional impact of RNF213 mutations, and exploring precision repair approaches. These innovative approaches offer novel insights into disease mechanisms and open new avenues for therapeutic intervention in MMD.

## Introduction

MMD was first reported in Japan in 1957 and officially named in 1969 [1], reflecting the “puff of smoke” appearance of collateral vessels seen on cerebral angiography. Initially thought to be endemic to East Asia, MMD has since been recognized worldwide [2],though genetic susceptibility and environmental risk factors exhibit considerable geographic and ethnic variation [3]. As a rare cerebrovascular disorder, MMD is primarily defined by aberrant vascular proliferation and remodeling at the skull base [1]. Its hallmark features include progressive stenosis or occlusion of the terminal portions of the internal carotid arteries and the proximal segments of the anterior and middle cerebral arteries [4, 5]. In response to chronic ischemia, fragile collateral networks develop compensatory to maintain cerebral perfusion. Neuroimaging, particularly angiography, remains indispensable for diagnosis [6], and a comprehensive differential diagnosis is essential to distinguish MMD from atherosclerotic cerebrovascular diseases and other vasculopathies with similar imaging findings.

Although precise pathogenesis is not well characterized, RNF213 mutations represent the foremost genetic risk factor in East Asian populations [7, 8]. While molecular mechanisms are still under investigation, emerging evidence suggests that these mutations contribute to cerebrovascular abnormalities by dysregulating endothelial cell (ECs) proliferation and migration [9–11]. Loss of RNF213 function has been implicated in pathological angiogenesis and impaired regulatory T-cell differentiation via K63-linked ubiquitination [12, 13],supporting the hypothesis that MMD may represent an immune-related angiopathy [14, 15]. However, progress in understanding MMD has been hampered by limited access to patient-derived lesioned vessels and the absence of animal models that fully recapitulate human disease pathology. Although current treatment strategies (e.g., surgical revascularization) effectively improve cerebral hemodynamics, they do not target the underlying molecular drivers. Conversely, iPSC-based disease models have emerged as promising tools for modeling MMD pathobiology, while CRISPR-mediated gene editing offers novel avenues for molecularly precise interventions [16, 17]. Notably, the recent FDA approval of CASGEVY™, the first CRISPR-based therapy for sickle cell disease, underscores the translational potential of genomic editing technologies [18].

In this review, we comprehensively examine the epidemiology, clinical manifestations, pathogenesis, and diagnostic approaches of MMD, establishing foundations for understanding its mechanistic basis. With a particular emphasis on RNF213-related pathways in MMD vasculopathy, we also highlight recent advances in patient-specific iPSC modeling combined with CRISPR-mediated genome editing, offering new insights into disease mechanisms and potential therapeutic strategies for MMD.

## Epidemiology of Moyamoya Disease

### Global Burden of Moyamoya Disease

MMD is a rare chronic cerebrovascular disorder exhibiting significant ethnic and regional variations. Epidemiological studies indicate that the majority of MMD cases occur in Asian populations, followed by Black and Caucasian/European groups [2]. However, recent analyses of U.S. inpatient databases suggest a trend toward more proportional racial distribution relative to the general population [19]. Despite this shift, due to genetic background differences, the overall prevalence remains substantially lower in Caucasian/European populations compared to East Asians, Likely due to underlying Genetic differences. Familial aggregation is well-documented in MMD, with family history reported in approximately 15% of patients in Japan and Korea [20–22], and 10.7% in China [23]. Relatives of affected individuals exhibit a significantly higher disease risk than the general population [24].Region-specific epidemiological data further highlight pronounced ethnic disparities: prevalence estimates are 10.5 per 100,000 in Japan [25], 3.92 per 100,000 in China [26], 16.1 per 100,000 in South Korea [27], and 2.2 per 100,000 in Washington state and California [2]. Notably, surveillance data indicate marked temporal increases in reported prevalence. Japan’s rate tripled from 3.16 per 100,000 in 1995 to 10.5 in 2007, while South Korea’s more than doubled between 2005 and 2011 [20, 25, 27]. This upward trend likely reflects improved diagnostic capabilities through non-invasive imaging and expanded healthcare access, rather than purely biological factors.

MMD imposes considerable socioeconomic burdens, with U.S. hospitalization costs averaging $133,754 per case—particularly for hemorrhagic presentations requiring surgical intervention [19]. High-prevalence countries such as Japan and South Korea face growing national Healthcare challenges due to rising incidence and improved long-term survival. Moreover, the disease entails significant neurological risks, with annual stroke recurrence rates ranging from 3.2% to 15% under conservative management [28]. Long-term outcomes remain suboptimal, particularly for hemorrhagic-onset cases, which show markedly worse survival compared to ischemic subtypes.

### Bimodal Age Distribution and Sex-Specific Patterns

MMD exhibits a consistent bimodal age distribution across diverse populations [29], characterized by one incidence peak in the first decade of life and another in the fourth decade [30, 31]. Epidemiological studies from Japan confirm this pattern, with peaks observed between ages 5-9 and 35-39 [25]. Similarly, data from China (2010) and the United States (Washington State and California, 2005) also reflect a bimodal distribution. While the childhood peak is consistent across these regions (ages 5-9), the adult peak occurs earlier in China (35-39 years) compared to the United States (55-59 years), and overall prevalence remains higher in China [26, 32, 33].

Sex-based disparities have also been reported. Studies from the United States, Japan, and other East Asian regions indicate a female predominance, with a female-to-male ratio ranging from approximately 1.8:1 to 2.2:1 [27]. In contrast, Chinese populations show no significant overall gender difference [32]; However, a notable sex-specific variation exists in the timing of onset: Chinese females demonstrate an earlier peak onset age compared to males [26].This suggests that although gender distribution may appear balanced at the population level, sex-related factors likely influence disease chronology. The earlier presentation among Chinese females raises important questions regarding potential roles of biological, hormonal (e.g., estrogen), or genetic factors that may accelerate vascular changes or hemodynamic instability in females.

Collectively, these epidemiological characteristics—including pronounced ethnic stratification, dynamic demographic trends, distinctive age and sex distributions, and considerable familial aggregation—underscore the intricate interplay between genetic susceptibility and population-specific factors in the pathogenesis of MMD.

### Genetic Loci and Polygenic Risk in Moyamoya Disease

Previous whole-genome analyses have identified several Genetic loci associated with MMD, including 3p26-p24 [34, 35], with subsequent studies implicating additional loci on 8q23 [36] and 17q25 [37, 38] in familial cases. Among these, the RNF213 Gene at 17q25 has been established as the major susceptibility gene in East Asian populations, as confirmed by genome-wide association studies and exon sequencing [34, 39, 40]. The p.R4810K variant in RNF213 represents the most common Genetic alteration, accounting for approximately 95% of familial and 80% of sporadic MMD cases in East Asians [41, 42].This association has been consistently replicated in case-control studies involving Japanese, Korean, and Chinese patients [43–47]. Liao et al. demonstrated that the p.R4810K variant significantly elevates the risk of familial MMD across these populations [48]. One study of 170 Chinese MMD patients identified 22 cases with the p.R4810K mutation [44], while another investigation of 260 patients revealed that 71.2% had no RNF213 mutation, 20.0% were heterozygous for p.R4810K, and 8.8% carried other rare or de novo variants [43].Consequently, screening for the p.R4810K variant may facilitate early identification of asymptomatic individuals, particularly those with a family history of MMD.

Although the p.R4810K mutation is undetectable in non-Asian MMD cohorts [49], emerging evidence has revealed population-specific rare variants in RNF213 among these patients, including p.R4019C, p.S4118F, and p.P4608S [50–52], the identified variants consistently localize to RNF213's C-terminal region. Unlike East Asian populations where p.R4810K represents the predominant mutation, European MMD cases are characterized by distinct C-terminal variants, implying ethnic-specific pathogenic mechanisms. Additionally, in a cohort study of 108 predominantly non-East Asian (European-descent) sporadic MMD patients, approximately 5-6% harbored rare pathogenic DIAPH1 variants [51]. Notably, all identified DIAPH1 mutations occurred exclusively in non-Asian populations, while RNF213 remains the primary genetic determinant of MMD in East Asia [53]. These genetic findings illustrate an important pathophysiological paradigm: although population-specific variants in RNF213 exhibit mutational diversity, they appear to converge on a common molecular mechanism—functional impairment of RNF213-mediated vascular homeostasis. This consolidates the role of RNF213 as a central molecular node in MMD pathogenesis across ethnicities, with additional susceptibility genes such as DIAPH1 contributing in specific populations. Elucidating this genotype-phenotype relationship not only advances our understanding of the genetic architecture of MMD but also underscores the importance of functional studies to uncover conserved disease mechanisms across diverse populations.

## Clinical Spectrum and Vascular Pathology of Moyamoya Disease

**Fig. 1:**
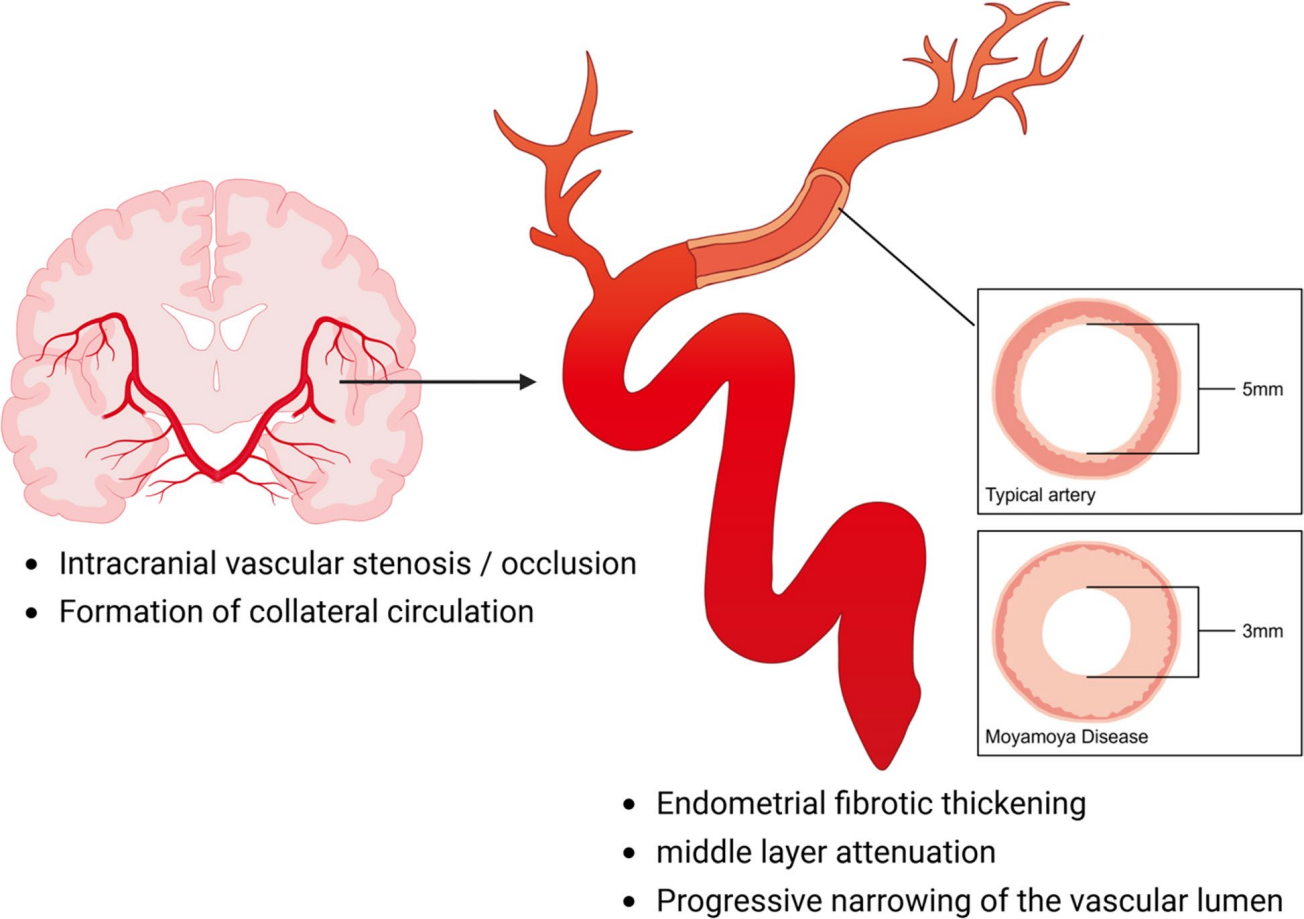
Pathological and vascular phenotypes in Moyamoya disease (created by BioRender, license VC28MP23H2). Cross-section of an affected intracranial artery reveals the underlying tissue changes: endometrial fibrotic thickening and middle layer attenuation with a thinned media. These changes drive the progressive narrowing of the vascular lumen, ultimately leading to the steno-occlusive events

MMD derives its name from the Japanese term "moyamoya," meaning "puff of smoke," which describes the characteristic angiographic appearance of the fine collateral vessels that develop in response to steno-occlusive changes. Clinically, MMD presents with transient ischemic attacks (TIAs), ischemic or hemorrhagic stroke, seizures, headaches, and cognitive impairment [30, 54]. Symptom profiles and incidence rates exhibit age-dependent variations across both East Asian and American populations [19]. The vascular pathology of MMD involves two primary components: (1) stenosis or occlusion at the terminal portion of the internal carotid artery (ICA), stimulating the development of compensatory collateral networks; and (2) involvement of posterior circulation vessels, including the posterior cerebral artery (PCA) and basal reticulate vessels, in a subset of cases [7]. These changes lead to progressive arterial narrowing and eventual occlusion (Fig. 1), impairing cerebral perfusion and resulting in two main clinical manifestations: cerebral ischemia and hemorrhage [55], which distinguish MMD from typical occlusive cerebrovascular disease. The anterior circulation—particularly the middle cerebral artery (MCA) and anterior cerebral artery (ACA)—is most frequently affected [56], commonly causing symptoms such as hemiparesis, aphasia, and unilateral sensory deficits [57]. Although PCA involvement was historically considered rare [58], recent evidence indicates that approximately 29% of MMD patients exhibit PCA stenosis, with 17% progressing to PCA infarction [59]. This occurs at similar frequencies in pediatric and adult patients and often emerges following revascularization surgery of the anterior circulation [60], suggesting its role as a marker of disease progression and poor prognosis. Furthermore, PCA stenosis has been genetically linked to the RNF213 c.14576G > A variant [47], a major risk allele in East Asian populations.

In pediatric patients, cerebral ischemic events—including TIAs, cerebral infarction, and cognitive decline—are the predominant manifestations [61, 62]. Symptoms often recur and may alternate sides due to the bilateral nature of the disease. Additional features include headaches [63], seizures, and involuntary movements [64]. Ischemic episodes are frequently triggered by hyperventilation, crying, stress, fatigue, or infection [65]. Progressive cerebral infarction can lead to permanent neurological deficits and cognitive impairment [66–68]. Headaches occur in approximately 20−22% of pediatric cases [54, 69], likely resulting from reduced cerebral blood flow and impaired vascular reserve. Notably, revascularization surgery may improve perfusion and alleviate headache symptoms [70].

In adults, cerebral hemorrhage becomes more common, primarily due to rupture of fragile collateral vessels or associated microaneurysms [55]. Other manifestations include seizures, cognitive deficits, and somatic dysfunction. Ischemic and hemorrhagic strokes each account for nearly half of adult presentations [63], with Hemorrhagic events predominating in patients over 40 years of age. Bleeding typically occurs in anterior circulation territories, often in periventricular regions [5]. Adult patients also show higher rates of intraparenchymal, intraventricular, and subarachnoid hemorrhage compared to children, in whom intracranial hemorrhage is rare [65]. Cerebral microbleeds (CMBs) are detected in 28-46% of MMD patients and are associated with an increased risk of intraventricular hemorrhage [71–73].

## Multifactorial Pathogenesis of Moyamoya Disease

**Fig. 2:**
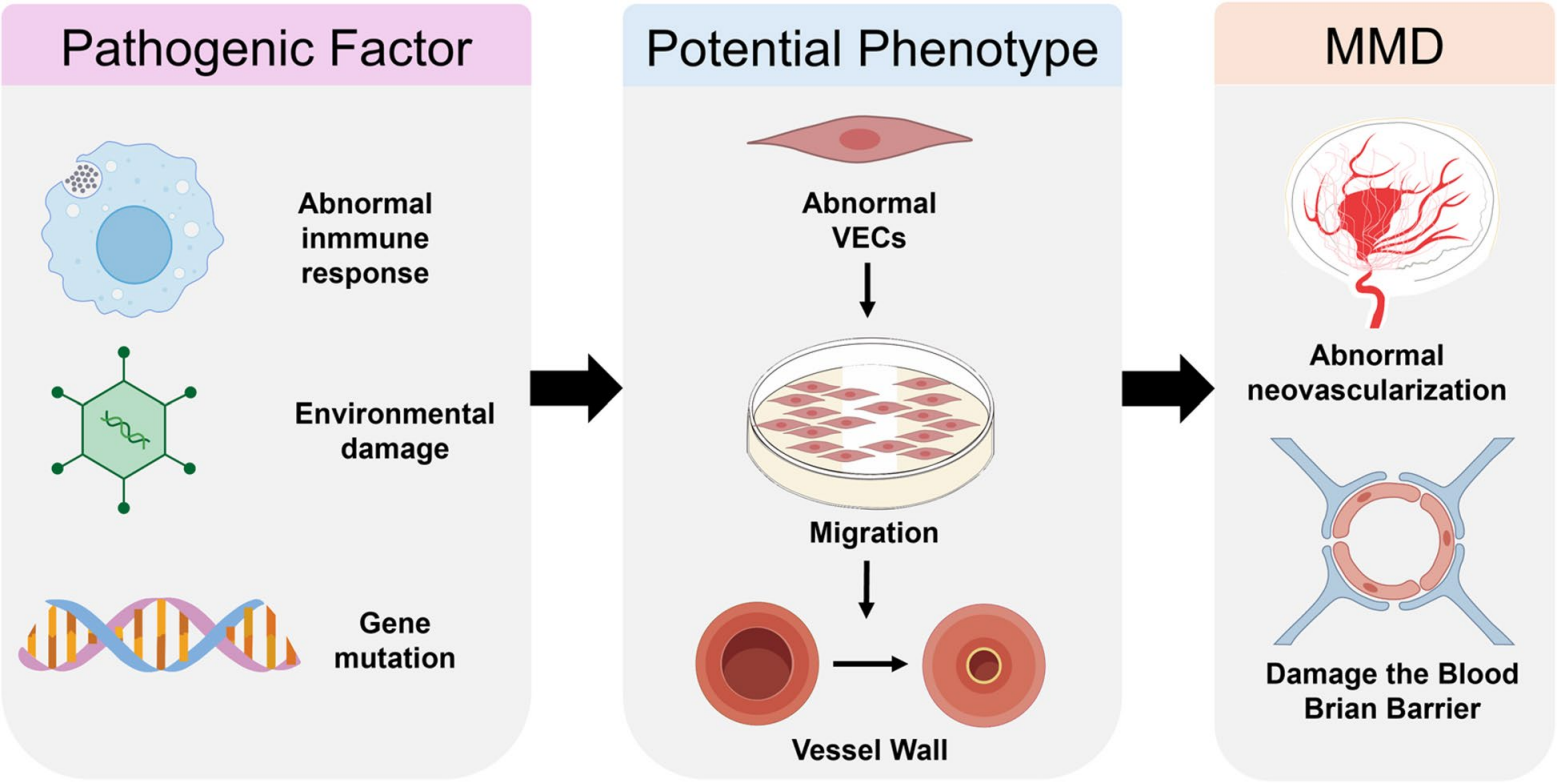
Key pathogenic mechanisms and pathological processes in Moyamoya disease. This diagram summarizes the current understanding of potential etiological factors contributing to Moyamoya disease. Pathogenic Factor is shown initiating a cascade that involves both genetic, immunological and environmental triggers. Potential phenotype of abnormal VECs is indicated, leading to the characteristic pathological changes in the vessel wall, such as intimal hyperplasia and luminal stenosis. This integrative model visualizes how multiple pathways may interact to cause the typical vasculopathy

Current treatments for MMD focus on symptomatic management and do not halt disease progression, underscoring the need to elucidate its underlying mechanisms. Although the precise etiology remains incompletely defined, emerging evidence implicates genetic predisposition, dysimmune responses and environmental factors in disease development (Fig. 2).

### RNF213-Centric Molecular Pathways and Polygenic Contributions

Genetic factors represent the most well-characterized component of MMD pathogenesis. The RNF213 gene, which encodes a large E3 ubiquitin ligase with Ring finger and AAA + ATPase domains, exhibits substrate-specific ubiquitination activity [74] and is implicated in protein degradation pathways affecting cerebrovascular development [75]. Ye et al. revealed that RNF213 knockout in endothelial cells activates YAP/TAZ signaling and upregulates VEGFR2, promoting pathological angiogenesis [13, 76]. A study demonstrated that the p.R4810K variant in RNF213 (predominant in East Asian populations) impairs angiogenic function and endothelial proliferation [77]. Xu et al. identified HAPLN3 as a genetic modifier that influences the penetrance of RNF213 p.R4810K [40, 78].

In addition to RNF213, other genes have also been implicated in MMD, including CCER2, ACTA2, BRCC3, and GUCY1A3 [79–82]. Genome-wide studies in Chinese cohorts have linked polymorphisms in PTPN11, GRB2, ITGB3, CBL, and HIF1A to MMD risk [83]. Some variants may elevate serum homocysteine levels [84],which promotes thrombosis and endothelial dysfunction [85]. Interestingly, patients with biallelic GUCY1A3 mutations often exhibit achalasia alongside MMD, though some present with isolated cerebrovascular pathology [82], highlighting the variable expressivity and genetic complexity of MMD.

### Dysregulated Epigenetic Landscapes in Moyamoya Disease

Accumulating evidence from multi-omics studies implicates that epigenetic dysregulation constitutes a critical mechanism in pathogenesis of MMD. A prominent aspect of this dysregulation is altered DNA methylation, which has been consistently observed in MMD patients. For instance, aberrant methylation in the promoter region of RNF213 has been shown to modulate its transcriptional activity, potentially influencing disease susceptibility [86]. Similarly, the promoter of SORT1 exhibits hypomethylation in endothelial colony-forming cells derived from MMD patients compared to healthy controls, which may contribute to abnormal vascular remodeling [87].Genome-wide methylation studies further reveal altered methylation profiles in the blood of ischemic MMD patients, affecting genes involved in cell proliferation and vascular function. He et al. identified distinct methylation signatures in MMD, including hypermethylation of STAB1, which may facilitate pathological crosstalk between endothelial and immune cells. Immunohistochemical analyses suggest that STAB1 promotes vascular endothelial thickening by enhancing extracellular matrix (ECM) synthesis [88, 89]. Meanwhile, Tokairin et al. reported reduced genome-wide methylation variability in MMD patients, indicating systemic epigenetic instability that may constrain adaptive transcriptional responses [90].

Another epigenetic mechanism dysregulated in MMD involves histone modifications. Specifically, reduced H3K27 acetylation in MMD-derived ECs is linked to suppressed RALDH2 expression and a consequent impairment in angiogenesis [91]. In parallel, a whole-exome sequencing identifies rare variants in chromatin remodeling genes such as CHD4, CNOT3, and SETD5 may disturb histone modification landscapes [92], thereby affecting MMD occurrence and development.

Furthermore, alterations in noncoding RNA (ncRNA) expression have also been identified in MMD. Dysregulation of miRNAs such as miR-126 and miR-let7c has been observed in serum, plasma, and cerebrospinal fluid of MMD patients [93–95], potentially affecting angiogenesis and inflammation through interactions with RNF213 or its downstream effectors. LncRNA (long noncoding RNA) are also differentially expressed in MMD blood samples and are implicated in inflammatory and angiogenic pathways [96]. Proteomic analyses of serum and cerebrospinal fluid (CSF) reveal alterations in ECM regulators (e.g., MMP-9), cytoskeletal proteins (FLNA, ZYX), and apolipoproteins involved in lipid metabolism, suggesting defects in vascular remodeling potentially mediated by post-translational mechanisms [97, 98]. circular RNA circZXDC is upregulated in MMD patients, particularly those carrying RNF213 mutations, and promotes a synthetic phenotype in vascular smooth muscle cells via the miR-125a-3p-ABCC6 axis, contributing to intimal hyperplasia [99].

Collectively, epigenetic dysregulation, encompassing DNA methylation, histone modifications and ncRNAs, may drive MMD pathogenesis via an upstream regulatory circuit that converges on RNF213.Technically, despite significant advances in mapping epigenomic landscapes through multi-omics approaches, current research remains largely correlative and is constrained by several limitations. There is a pronounced lack of causal validation and temporal resolution linking epigenetic changes to disease progression. Future studies must leverage multi-omics integration to transition from descriptive associations to mechanistic investigations that definitively establish the pathogenic contributions of specific epigenetic modifications in MMD.

### The Role of Inflammation and Immune Dysregulation in Moyamoya Disease

Current understanding of MMD pathogenesis increasingly revolves around two interconnected pathological processes: dysregulated inflammatory responses and aberrant thrombogenesis [100–102]. Multiple studies indicate that systemic inflammation plays a central role in MMD, characterized by elevated inflammatory cytokine secretion and altered immune cell populations—particularly among CD4^+^ T cells, CD8^+^ T cells, and B cells with peripheral blood mononuclear cells (PBMCs) of affected individuals [103–106]. Inflammatory activation promotes ECs proliferation and migration, contributing to vascular wall thickening and luminal narrowing [107], ultimately impairing vasodilation capacity. Evidence from RNF213 knockout mice suggests that impaired nitric oxide (NO) production in macrophages may underlie vascular dysfunction [108]. Mutations in RNF213 are thought to disrupt vascular homeostasis, in part through interference with NO signaling. This is further supported by the identification of biallelic loss-of-function mutations in GUCY1A3, which encodes the primary receptor for NO, in a subset of autosomal recessive MMD cases [108, 109].Concurrently, dysfunctional ECs release inflammatory and thrombotic factors that exacerbate vascular stenosis [110]. Elevated levels of oxidative stress markers, such as thiol-disulfide compounds and ischemia-modified albumin, have also been detected in PBMCs from MMD patients, suggesting a role for oxidative imbalance in disease progression [111]. Recent analyses of superficial temporal arteries (STA) from MMD patients revealed increased expression of TNF-α and CTLA4, reinforcing the involvement of inflammatory pathways in disease development [112]. Furthermore, chronic cerebral ischemia stimulates the release of growth factors like TGF-β, accelerating smooth muscle cell migration and proliferation, which leads to arterial stiffening and progressive stenosis [113].

Takeda et al. demonstrated that E3-deficient RNF213 mutants enhance NF-κB activation and promote apoptosis via their AAA + domains, which impairs the function of vascular endothelial cells (VECs) [114]. Additional studies in mouse models indicate that Rnf213 knockout disrupts the Akt/GSK-3β/β-catenin/Bcl-2 axis, which is critical for regulating apoptosis [76]. Immune profiling of MMD patient PBMCs revealed significant alterations in innate immune cell subsets and functions, along with overactivation of the TLR/MyD88/NF-κB pathway across most innate immune cells. These changes suggest both impaired anti-infective responses and heightened inflammatory activation in MMD [115]. Bhardwaj et al*.* identified a hypoxia-responsive pathway involving PTP1B and ABL1/2 that regulates RNF213 in cancer cells [116], highlighting the pleiotropic roles of RNF213 at the intersection of vascular integrity and inflammatory regulation.

Beyond immune dysfunction, growing evidence implicates metabolic dysregulation as a key component of MMD, potentially forming a critical immune-metabolic axis. Metabolomic studies have further identified distinctive disturbances in MMD, including reduced circulating lysophosphatidylcholines(LPCs),altered branched-chain amino acid profiles, and dysregulated bile acid metabolism [117]. Together, these observations collectively underscore the interplay between immune and metabolic dysfunction in MMD. The identified metabolic disturbances, which correlate with inflammatory states, are not merely bystanders but may play a role in disease mechanisms. This nominates them as promising, clinically accessible diagnostic biomarkers worthy of further validation.

### Integrated Pathogenesis of Moyamoya Disease: Oxidative Stress, Matrix Remodeling, and Environmental Triggers

In addition to sustained inflammatory stimulation, oxidative stress and other factors represents another crucial mechanism driving endothelial dysfunction in MMD. It has been found that VECs are affected by oxidative stress. Elevated reactive oxygen species (ROS) inflict damage on VECs, impairing their ability to regulate vasodilation and constriction, and compromising vascular integrity—thereby facilitating the development of moyamoya collateral vessels. Studies have detected heightened oxidative stress in the peripheral blood of MMD patients, which promotes the release of inflammatory factors and exacerbates vascular injury, ultimately stimulating the proliferation and migration of vascular smooth muscle cells and contributing to stenosis and occlusion [111, 118]. MMPs are also implicated in MMD pathogenesis through degradation of the vascular extracellular matrix. By breaking down collagen and elastin fibers within the vessel wall, MMPs weaken structural integrity [119], leading to vascular dilation and distortion that underlies the characteristic abnormal network at the brain’s base. Furthermore, MMPs influence vascular remodeling by modulating the extracellular environment to affect the migration and proliferation of both vascular smooth muscle cells (VSMCs) and ECs [120–122]. Chronic cerebral hypoperfusion in MMD creates a hypoxic microenvironment, resulting in elevated HIF-1α levels in the intima of middle cerebral arteries [123]. HIF-1α activation promotes angiogenesis via upregulation of VEGF expression—a compensatory mechanism that, in MMD, yields structurally and functionally deficient vessels [124, 125].ET-1, a potent vasoconstrictor, has been shown to impair microvascular and conduit vessel function [126], suggesting its potential role as a therapeutic target in MMD.. Notably, only approximately 0.5% of individuals carrying RNF213 mutations develop MMD, implying that epigenetic mechanisms—such as DNA methylation—or environmental triggers like chronic inflammation or recurrent infections may act synergistically with genetic susceptibility to precipitate the disease. These multifactorial interactions drive progressive vascular stenosis and aberrant collateral formation, which represent core pathological hallmarks of MMD. A recent study proposed a link between infection and MMD onset, suggesting that while RNF213 mutations impair ubiquitination capacity, exposure to bacterial lipopolysaccharide (LPS) or other infectious agents may serve as ubiquitination substrates that activate mutant RNF213 and trigger disease in genetically susceptible individuals [127].

Transcriptomic analyses further support the role of metabolic and mitochondrial dysfunction in MMD. Vascular tissues from MMD patients show downregulation of mitochondrial-related genes [128], which not compromises energy production but also increases ROS generation, promoting apoptosis and cellular dysfunction [129]. Similarly, PBMC studies reveal upregulation of hydrogen peroxide metabolism, indicating systemic oxidative stress [130]. Additionally, mitochondrial impairments in peripheral T cells—marked by defective oxidative phosphorylation, elevated endoplasmic reticulum stress, and altered immune checkpoint expression (e.g., PD-1, ICOS)—suggest profound immunometabolism dysregulation that may contribute to vascular inflammation and remodeling [131]. A deeper understanding of these pathways may reveal novel therapeutic strategies—such as enhancing mitochondrial function or modulating oxidative phosphorylation could restore immune homeostasis, alleviate vascular inflammation, and open new avenues for treatment.

**Fig. 3:**
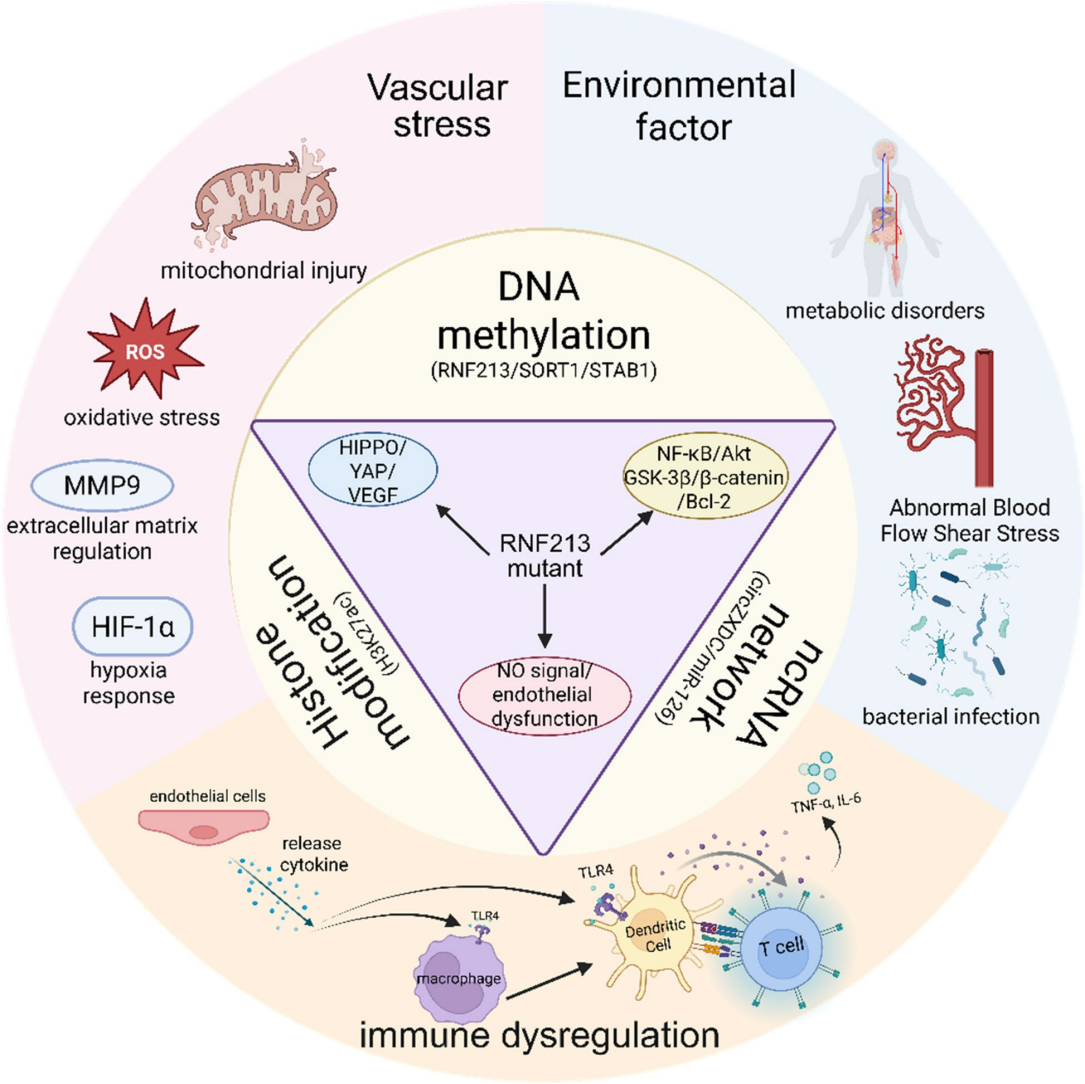
An Integrated Pathogenic Schema of Moyamoya Disease (created by BioRender, license HJ28MVXEMS). This schematic synthesizes key drivers of Moyamoya disease pathogenesis into a comprehensive mechanistic network, encompassing genetic, immune, hemodynamic, and metabolic factors

Collectively, the pathogenesis of MMD is now recognized as a complex, multifactorial process involving dynamic interactions between genetic susceptibility, epigenetic regulation, inflammatory activation, and environmental influences. In particular, the RNF213 gene serves as a major genetic determinant, though its penetrance is modulated by additional polygenic contributors and epigenetic mechanisms such as DNA methylation and non-coding RNA activity. Concurrently, innate and adaptive immune dysregulation drives sustained vascular inflammation, which synergizes with oxidative stress, extracellular matrix remodeling, and exogenous triggers to impair vascular integrity and promote aberrant angiogenesis. Rather than operating in isolation, these pathways converge to disrupt cerebral vascular homeostasis, ultimately culminating in the characteristic steno-occlusive changes and compensatory collateral vessel formation that define MMD (Fig. 3). A comprehensive understanding of these integrated mechanisms is essential for the development of targeted diagnostic and therapeutic strategies.

## Modeling Moyamoya Disease: Challenges in Phenotypic Recapitulation and the Rise of Patient-Derived Organoids

To investigate the underlying mechanisms and progression of MMD, researchers have developed various experimental models, primarily through manipulation of RNF213, to simulate disease phenotypes. However, replicating the hallmark feature of MMD, namely the characteristic “smoky” vascular network at the base of the brain, remains a major challenge. Most gene-edited animals or cerebral ischemia models fail to fully recapitulate these pathological features (Table 1). Furthermore, the limited availability of intracranial arterial tissue from MMD patients continues to constrain deeper mechanistic exploration. In vivo studies have underscored that the etiology of MMD involves a complex interplay between genetic susceptibility and environmental triggers.

**Table 1:** The reasons for the model organisms used in the past unable to simulate MMD models

Model Type		Disadvantages
**Animal Model**	**Zebrafish**	Lack similarities in vasculature with mammals [132]
	**Mice and Rats**	Exhibit varying cerebrovascular organization
		Lack communicating arteries within the circle of Willis [133]
		Severely reducing the tolerance to infarcts
	**Rabbit**	Long-term maintenance requirements limit widespread adoption. [134]
		Failed to successfully reappear typical progression
	**Cat**	The stenotic changes and development of moyamoya vessels were not expounded [135]
	**Monkey**	Neither stenosis of ICAs nor moyamoya vessels was observed by an intravenous repeated injection of MDP
	**Dog**	failure to identify intracranial immune complexes limited its pathogenic implication [136]
		Vascular anatomy does not perfectly match human conditions. [137]
	**Miniature Pig**	Unable to evaluate the cerebrovascular. [138]
**In vitro models**		Tissue-level complexity and long-term pathophysiological context
**Organoid Models**		Lack the complex in vivo microenvironment

### Animal models

To elucidate the mechanisms and progression of MMD, researchers have developed various experimental models, primarily centered on RNF213 manipulation. Initially, Liu et al*.* [39] established an RNF213-knockdown zebrafish model that displayed abnormal cranial angiogenesis. Subsequently, Wen et al*.* [139] generated an RNF213 mutant zebrafish model using the transcription activator-like effector nuclease (TALEN) technique, observing aberrant intervessel and secondary cerebral angiogenesis, as well as circulatory defects marked by abnormal vascular buds in the trunk and head. Roberts et al*.* [140] developed a mouse model by surgically implanting microcoils (0.16 mm in diameter, 1.5 mm in length) into the ICA, inducing stenosis and hypoperfusion that elicited vascular changes resembling MMD. In the study by Sonobe et al*.* [122], mice with an exon 32 deletion in RNF213 failed to develop basal cerebral vascular abnormalities, suggesting that genetic predisposition alone is insufficient and secondary insults are critical for disease manifestation [141]. In the same year, Sonobe et al*. *[142] also demonstrated that following carotid artery ligation in RNF213 knockout mice, there was a marked increase in MMP-9 expression, along with thinning of the vascular wall, consistent with the finding of vascular remodeling in MMD and reflecting early-stage pathological changes characteristic of the disease. Kanoke et al*.* [143] observed that RNF213 mutant mice had reduced regulatory T-cell populations, potentially disrupted immune tolerance and facilitated MMD onset. After ischemic challenges such as transient MCAO and femoral artery ligation, these mutants show enhanced angiogenic recovery compared to wild-type mice [144]. However, neither the RNF213-R4828K knock-in model [133], nor mice overexpressing the R4757K variant under hypoxia exhibited significant pathological angiogenesis [145], indicating limitations in recapitulating the full MMD phenotype.

Additional models include the bilateral common carotid artery stenosis (BCAS) model in RNF213-knockout mice established by Morimoto et al*.* [146] to simulate chronic hypoperfusion. Rabbit models induced through allogeneic serum injection followed by antigen or antibody challenge demonstrated pronounced vascular proliferation, supporting an immune-mediated mechanism reminiscent of allergic angiitis [134]. Kamata et al*.* [135] attempted to build a cat model uniting an immune reaction and an arterial occlusion technique, and they found the intimal changes of ICAs and their branches bilaterally. Similarly, Nakamura et al*.* [138] used internal carotid artery occlusion (ICAO) in miniature pigs to show that functional revascularization requires specific hemodynamic and environmental stimuli.

Despite these efforts, no animal model fully replicates the characteristic “moyamoya” collateral network, underscoring the necessity for more sophisticated systems that integrate genetic, immunological, and hemodynamic factors. Consequently, there remains a pressing need for improved models to fully dissect MMD pathogenesis. Nonetheless, existing models remain valuable for probing gene-environment interactions, tracking disease progression, and evaluating interventions. Future directions may include multi-gene editing, combined immune modulation, and advanced hemodynamic simulations to enhance clinical relevance and reproducibility. Moreover, models based on other MMD-associated genes have emerged. Starosolski et al*.* [147] reported that ACTA2 knockout mice exhibit cerebral vascular thinning and narrowing, while Ren et al. [148] demonstrated that NEO1 knockout models display similar cerebrovascular phenotypes, closely mirroring aspects of human MMD pathology.

Although animal models have substantially advanced our understanding of genetic susceptibility, immune mechanisms and hemodynamic contributions in MMD, they consistently fail to recapitulate the defining “moyamoya” vascular collateral network—particularly within the basilar region. These limitations have accelerated a shift toward more human-relevant experimental platforms, such as in vitro cell models and brain organoids, which provide greater precision for investigating disease mechanisms and screening potential therapeutics in a human-specific context.

### In vitro models

#### Conventional Cell models

In vitro models are increasingly recognized as supplemental alternatives to animal models, providing enhanced experimental control and scalability. For instance, ECs models stimulated with serum from MMD patients have revealed critical vasomodulatory effects: MMD serum promotes angiogenesis, cytoskeletal reorganization, and upregulation of FLNA and ZYX via AKT signaling, recapitulating early vascular remodeling processes and highlighting potential diagnostic biomarkers and therapeutic targets [97]. Additionally, serum amyloid A2 (SAA2) has been found upregulated in MMD patient serum. In vitro studies demonstrate that SAA2 induces phenotypic changes in VSMCs associated with intimal hyperplasia in cerebral vessels affected by MMD [149], suggesting its utility as a biomarker for diagnosis or progression monitoring. He et al. [150] identified several exosome circRNAs (e.g., IPO11, PRMT1, CACNA1F) enriched in MMD, functioning as mobile epigenetic regulators of angiogenesis and occlusion.

These in vitro systems offer considerable benefits in usability and scalability, supporting both mechanistic exploration and biomarker identification. However, they remain limited in their ability to fully replicate the architectural complexity of native tissues and the chronic, progressive nature of MMD pathology. They are particularly useful for elucidating signaling pathways, identifying circulating and exosomal biomarkers, and conducting preliminary drug screening. To better approximate human disease conditions, future improvements could involve incorporating multicellular co-cultures, integrating immune components, and simulating mechano-fluidic microenvironments.

#### iPSC-derived vascular models

To bridge the gap between conventional in vitro models and human pathophysiology, subsequent studies have employed iPSC technology. Researchers subsequently generated iPSC-derived vascular organoids from MMD patients, revealing that RNF213 mutations lead to impaired vascular branching and dysfunction. Vascular organoids have recently emerged as a powerful experimental platform to address long-standing challenges in vascular disease research. By integrating multi-omics approaches (e.g., proteomics, epigenetics, metabolomics), CRISPR-based technologies, and drug screening, they provide a novel framework for investigating disease mechanisms and potential therapies for cerebrovascular disorders. Conventional models face inherent limitations: animal studies often lack human genetic context, while cell-based systems fail to recapitulate tissue- and organ-level pathology. In this context, vascular organoids represent a promising alternative. He et al*.* [98] successfully generated vascular organoids derived from iPSCs from patients with MMD and matched controls. Using single-cell sequencing and proteomic analyses, they demonstrated that overexpression of TUBA and TUBB drives the transformation, proliferation, and migration of VSMCs, ultimately leading to intimal hyperplasia. These findings provide the most recent organoid-level evidence clarifying the role of VSMCs in MMD pathogenesis. Derived from patient-specific iPSCs, MMD vascular organoids retain the full genetic background of the donors, offering a significant advantage over conventional animal models (e.g., surgical ligation or gene-knockout approaches), which often lack human genetic context and fail to fully replicate disease-specific pathology. However, current organoid models do not fully incorporate the complex in vivo microenvironment, including immune interactions and systemic influences. Further validation through transplantation into animal hosts will be essential to assess their physiological relevance and bridge the gap between in vitro findings and in vivo disease mechanisms.

## Diagnostic Strategies and Criteria for Moyamoya Disease

Diagnosis of MMD is primarily based on the identification of the characteristic small, tortuous “moyamoya vessels” on angiography, supplemented by evidence of ischemic lesions on MRI and reduced cerebrovascular reserve on perfusion studies. Clinical evaluation of MMD requires a systematic approach encompassing detailed history-taking (focusing on demographic factors and symptom progression), comprehensive neurological examination (assessing motor, speech/swallowing, and cognitive functions), and confirmatory ancillary investigations (including vascular imaging and hemodynamic studies) to establish diagnosis and guide management. This standardized diagnostic framework is essential to address the heterogeneous and progressive nature of MMD, ensuring accurate classification and tailored management.

### Clinical Diagnostic Approaches

MMD characterized by bilateral steno-occlusion of the terminal internal carotid arteries and compensatory collateral vasculature, demands a multimodal neuroimaging approach to reconcile its diagnostic complexity and therapeutic implications. DSA remains the gold standard for delineating pathognomonic features—progressive supraglenoid ICA stenosis and the "puff of smoke" collateral network-with superior spatial resolution [151]. Non-invasive modalities, including magnetic resonance angiography (MRA) and computed tomography angiography (CTA), provide valuable alternatives for high-resolution vascular assessment [152], enabling detailed evaluation of arterial stenosis and collateral formation [153].Sugino et al*.* reported that CTA outperforms MRA in detecting very small stenotic lesions in the ICA and MCA [152], while MRA is widely utilized as a completely non-invasive technique that avoids contrast agents and radiation exposure [154]. Perfusion imaging plays a critical role in functional assessment. Techniques such as arterial spin labeling (ASL) MRI and single-photon emission computed tomography (SPECT) allow quantification of cerebral blood flow (CBF) and cerebrovascular reactivity (CVR), which are essential for predicting ischemic and hemorrhagic risk, as well as postoperative outcomes [155]. Positron emission tomography (PET) has also emerged as a robust tool for evaluating cerebral hemodynamics and metabolism, employing tracers such as C^15^O for cerebral blood volume, H_2_^15^O for CBF, and^15^O_2_for oxygen extraction fraction (OEF). Conventional MRI and MRA are instrumental in early screening and diagnosis. T2-FLAIR sequences can reveal ischemic tissue changes [156], while MRA can show intracranial aortic stenosis and compensatory collateral circulation. Furthermore, advanced MRI techniques, including blood oxygen level-dependent (BOLD) MRI for CVR assessment [157] and dynamic susceptibility contrast (DSC) MRI for perfusion analysis [155], have been explored in MMD imaging. The integration of these multimodal imaging techniques is essential for achieving diagnostic accuracy, monitoring disease progression, and informing clinical management in MMD.

### Established Diagnostic Criteria

The definitive diagnosis of MMD relies on the identification of characteristic angiographic features alongside the exclusion of other systemic or inflammatory vascular disorders that may mimic its presentation. Diagnostic criteria require bilateral steno-occlusive changes involving the terminal ICAs and the proximal segments of the ACA and MCA, accompanied by the development of abnormal basal collateral vessels that produce the classic “puff of smoke” appearance on angiography.

Due to overlapping clinical and radiographic features, MMD must be carefully distinguished from other vascular disorders [31], such as intracranial atherosclerosis, cerebral vasculitis, and fibromuscular dysplasia (FMD).Intracranial atherosclerosis typically affects older individuals and is characterized by focal atherosclerotic plaques and irregular luminal narrowing, often with a dispersed arterial distribution [158]. In contrast, MMD demonstrates symmetric steno-occlusion localized to the terminal ICA and proximal cerebral arteries. Cerebral vasculitis, resulting from inflammatory endothelial injury, often presents with multifocal stenosis, segmental wall thickening, and contrast enhancement [159], whereas MMD exhibits non-inflammatory bilateral terminal ICA involvement with prominent basal collateralization. FMD classically displays a “string-of-beads” appearance in medium-sized arteries due to alternating stenosis and dilation [160], typically sparing the terminal ICA and lacking moyamoya-type collateral networks.

A conclusive diagnosis of MMD necessitates angiographic confirmation of bilateral terminal ICA and proximal cerebral artery stenosis, evidence of basal moyamoya vessels, and the absence of markers suggestive of inflammatory or systemic vascular diseases. Given the potential imaging overlap among these conditions, accurate differential diagnosis requires a multimodal approach incorporating vascular and perfusion imaging. This integrated strategy enables precise characterization of the progressive steno-occlusive changes and compensatory collateralization typical of MMD, thereby supporting appropriate clinical management. The current diagnostic criteria for MMD are summarized in Table 2, with DSA remaining the most accurate imaging standard.

**Table 2:** Key Diagnostic Modalities for Moyamoya Disease

Assessment	Category	Content
Clinical Assessment	History Collection	Basical information: Gender, age, race, pedigree, duration of the disease, onset manner, symptoms and severity, and progression
	Physical Examination	Muscle strength, tone, tremors, abnormal gait, ataxia, dysarthria, swallowing function, speech functions, etc
		The National Institutes of Health Stroke Scale (NIHSS) score and the modified Rankin scale score
		Cognitive function and personality changes should be evaluated
Radiological Examination	Digital Subtraction Angiography (DSA)	The "gold standard" for imaging diagnosis of MMD [161]
	CT Angiography (CTA) or Magnetic Resonance Angiography (MRA)	The main methods for screening and evaluating MMD [162]
	Cerebral CT Perfusion Imaging (CTP) and Magnetic Resonance Perfusion Imaging	Dynamic susceptibility contrast-enhanced MRI and arterial spin labeling [163]

Beyond conventional imaging, novel molecular diagnostic strategies are increasingly contributing to the understanding of MMD. The MOYAOMICS project—a large-scale multi-omics initiative—has identified several candidate biomarkers linked to MMD pathogenesis and progression. Advanced techniques, such as single-cell RNA sequencing and mass cytometry, are being used to investigate cellular heterogeneity in peripheral blood samples from MMD patients [164]. Concurrently, radiomics-based image analysis supports early detection, phenotypic characterization, and longitudinal monitoring of MMD-related cerebral alterations. Metabolomic investigations have further revealed that specific biomarkers, notably elevated glutamine levels, are independently correlated with stroke risk in MMD [165]. This metabolomic approach not only offers potential for improving diagnostic precision but also provides mechanistic insights into disease pathways.

These developments indicate that integrating advanced imaging with multidimensional biomarker profiling could significantly enhance early detection, risk stratification, and personalized therapeutic strategies for MMD in the future.

### The Suzuki Staging System

The Suzuki staging system, initially introduced in 1969 by Japanese scholars Suzuki Jiro and Takaku Akira, remains the most widely used imaging-based classification system for assessing cerebrovascular progression in MMD [41]. Based primarily on DSA findings, this system methodically outlines the evolution and regression of the ICA system and its collateral circulation, categorizing the disease into six distinct stages.

This staging framework captures the dynamic pathological continuum of MMD—from initial ICA stenosis to complete occlusion, the proliferation and subsequent diminution of moyamoya vessels, and the eventual development of compensatory collateral supply from external carotid artery branches. Clinically, stages II to IV are regarded as the optimal window for surgical revascularization, such as superficial temporal artery–middle cerebral artery (STA-MCA) bypass. In contrast, advanced stages (V-VI) signify late disease, marked by an elevated risk of complications including intracranial hemorrhage, cerebral atrophy, and cognitive impairment [166].

Beyond its diagnostic relevance, the Suzuki staging system offers essential guidance for clinical decision-making, surgical timing, and prognostic assessment. When integrated with contemporary hemodynamic and functional imaging modalities, it continues to serve as a cornerstone in the comprehensive evaluation and management of moyamoya disease.

## Therapeutic Interventions of Moyamoya Disease

### Established Standard Treatments

MMD is managed through either conservative pharmacological therapy or surgical revascularization, depending on disease severity and patient eligibility. Conservative therapy, typically reserved for non-surgical candidates, aims to alleviate symptoms and prevent cerebral thrombosis but does not alter disease progression and lacks robust clinical evidence regarding its efficacy [167]. Antiplatelet agents such as aspirin are commonly used in chronic ischemic or acute cerebral infarction subtypes to reduce microthrombotic risk, though their application is limited in asymptomatic or hemorrhagic presentations due to potential bleeding complications. Another conservative agent is calcium channel blockers, like nimodipine, which selectively dilate cerebral vessels, lower stroke incidence and improve postoperative outcomes in pediatric ischemic cases. However, medication need cautious dosing to avoid hypotension. Statins may support angiogenesis and neuroplasticity by mobilizing endothelial progenitor cells, while neuroprotective agents help alleviate neurological symptoms through mechanisms such as free radical scavenging and inhibition of lipid peroxidation. Despite these benefits, conservative management remains palliative, underscoring the need for surgical intervention in eligible patients.

Surgical revascularization represents the cornerstone of definitive MMD treatment, aimed at improving cerebral perfusion by establishing collateral circulation from the external carotid artery system [41]. Direct revascularization provides immediate hemodynamic improvement by directly anastomosing extracranial and intracranial arteries. However, these procedures are technically demanding, require temporary occlusion of recipient vessels, and carry risks such as hyperperfusion syndrome. Indirect revascularization facilitates neovascularization by introducing angiogenic tissue to the brain surface without the need for temporary MCA occlusion. Although collateral development occurs over weeks to months, indirect techniques are considered safer and are often preferred in pediatric or anatomically complex cases [168].Combined revascularization integrates both direct and indirect approaches to optimize cerebral blood flow, though it involves more extensive surgical exposure and heightened technical challenges [169]. The selection of surgical strategy depends on patient age, disease stage, Suzuki angiographic grade, and surgical expertise. While direct bypass offers rapid hemodynamic correction, indirect methods prioritize safety and gradual revascularization. Ongoing advancements focus on refining minimally invasive techniques, improving patient selection criteria, and individualizing surgical planning to enhance functional outcomes in this complex disorder. A summary of current treatment options for MMD is provided in Table 3.

**Table 3:** Current Treatment Paradigms for Moyamoya Disease

Treatment Approaches		Advantages	Disadvantages	
**Surgical Treatment** [41]	Direct Blood Revascularization Surgery	Immediately increase blood flow to ischemic brain tissue [167]	•Require craniotomy •Establishment of vascular access and direct vascular manipulation require a larger surgical field of view •Non-minimally invasive surgery	
		Rapidly improve hemodynamic status		
	Indirect Blood Revascularization Surgery	Does not require temporary occlusion of the middle cerebral artery branches		
		Relatively simple operation suitable for children and patients with complex conditions [168]		
	Combined Blood Revascularization Surgery	Combines the advantages of direct and indirect blood revascularization surgery		
**Conservative Treatment** [167]	Antiplatelet Agents	Prevent the formation of microthrombi	Aspirin may increase the risk of intracranial hemorrhage	•Can only alleviate the phenotype •Lack clinical evidence on curative effect
	Calcium Channel Blockers	Selectively dilates cerebral blood vessels	High dose may lead to hypotension	
		Improves postoperative clinical function in children with ischemic MMD	Reduce cerebral perfusion	
			Exacerbating patients' ischemic hypoxia symptoms	
	Statin Drugs	Mobilize bone marrow endothelial progenitor cells		
		Enhance neuroplasticity		
		Promote angiogenesis		
	Neuroprotective Drugs	Edaravone can remove free radicals		
		Inhibit lipid peroxidation		
		Relieve neurological symptoms		

Since the 1970 s, direct revascularization has been widely utilized as a surgical treatment for MMD [170]. Between 2009 and 2013, South Korea observed a significant increase of 10,506 newly diagnosed MMD patients [171], during which a total of 3,326 revascularization surgeries were performed. Among these, 2,157 cases (64.8%) underwent direct or combined revascularization, while 1,169 cases (35.1%) received indirect revascularization. Long-term follow-up demonstrated that both adult and pediatric patients benefited from direct or combined revascularization, with a significant reduction in stroke risk [172–176]. The primary goal of MMD management is stroke prevention [169]. Studies indicate that the annual stroke incidence under conservative treatment ranges from 3.2% to 15% [177–180].In contrast, patients treated with direct bypass surgery experience markedly lower stroke rates—approximately 0.0% to 1.6% in adults (weighted average ~ 1.4%) [172, 181] and as low as ~ 0.2% in children [182–184].Indirect bypass is associated with higher stroke incidence, averaging 5.6% in adults [171, 174, 185] and 1.6% in children [186, 187]. Despite its efficacy, revascularization surgery carries inherent risks. Perioperative and postoperative complications including ischemic stroke (1.6%−16%) [179, 181], hyperperfusion syndrome (21.5%−50%) [188, 189], hemorrhagic stroke (0.7%−8.0%) [169, 181], and wound or scalp complications (17.6%−21.4%) [190, 191].These risks are influenced by patient-specific hemodynamics, surgical complexity, and postoperative management. Given these findings, early diagnosis and timely surgical intervention—particularly in pediatric patients—are crucial for reducing stroke incidence and optimizing long-term neurological outcomes in MMD.

### Emerging Therapeutic Strategies Targeting Molecular Pathways in Moyamoya Disease

Despite the possibility of isolating various primary cell types from living donors, certain cell types, such as brain vascular cells (primarily associated with MMD), remain largely inaccessible. Translational research employing stem cell-based disease models is critical for advancing therapeutic development. These biomimetic systems provide a powerful platform for verifying drug safety profiles, identifying optimal cell sources, elucidating essential molecular factors, and refining delivery strategies [192, 193]

#### iPSC-based Cell Therapy

iPSCs are a type of pluripotent stem cell generated from somatic cells (e.g., skin or blood cells) through the cell reprogramming [194]. iPSC-based therapies have emerged as promising strategies for treating complex rare diseases influenced by genetic and environmental factors, including neurodegenerative disorders [195, 196], cardiovascular diseases [197, 198], and diabetes [199]. The therapeutic approach typically involves reprogramming patient-derived somatic cells into iPSCs, which are then differentiated into relevant functional cell type (such as neurons, cardiomyocytes, or hepatocytes) for tissue repair or regeneration. For instance, in Parkinson’s disease research, midbrain dopamine neurons derived from iPSCs were sorted using the CORIN marker to enrich for transplantable progenitors with reduced tumorigenic risk. These CORIN^+^ cells, upon transplantation into a 6-OHDA-induced rat model, survived, matured into functional dopamine neurons, and ameliorated motor deficits without significant tumor formation [200]. Similarly, genetically engineered iPSC-derived cardiomyocytes have demonstrated therapeutic potential in murine models of myocardial infarction [201]. Beyond cell replacement, iPSCs also serve as scalable platforms for disease modeling and drug screening, exemplified by a genome-wide inducible CRISPR screen in iPSCs that identified neddylation as a regulator of neuronal aging and Alzheimer’s disease neurodegeneration [202].

Previous investigations utilizing iPSC-based models of MMD have yielded important pathophysiological insights. Hitomi et al*.* demonstrated that the tubular formation of vascular ECs was reduced using MMD patient-derived iPSC and overexpression of the RNF213 R4810K allele inhibited cell proliferation with HUVECs [77]. Other studies using MMD patient-derived iPSCs [203, 204] indicated that VSMCs showed comparable proliferation, migration, and contraction capabilities to healthy controls, suggesting that additional microenvironmental factors may be necessary to elicit pathological behavior. Kang et al. [205] demonstrated that smooth muscle progenitor cells (SPCs) from MMD patients formed disorganized vascular structures and displayed altered expression of genes related to vascular development, while iPSC-derived ECs from familial MMD cases did not exhibit enhanced proliferation. However, MMD-derived ECs exhibited distinct protein profiles versus controls, featuring impaired angiogenic capacity and upregulated mRNA splicing factors potential pathogenic contributors to disease initiation [206]. These in vitro findings sometimes diverge from clinical observations, underscoring the importance of incorporating multicellular interactions and physiological contexts into disease models. Moreover, stem cell therapies utilizing mesenchymal stem cells (MSCs) or neural stem/progenitor cells (NSCs) have also been explored for stroke treatment, aiming to promote both neuroprotection and neural repair [207]. iPSC-based approaches have been successfully applied to model numerous rare diseases such as Leigh syndrome (LS) [208], Kearns Sayre syndrome (KSS) [209],Catecholaminergic polymorphic ventricular tachycardia (CPVT) [210] [211], etc*.*, which can faithfully recapitulate human biology and found potential targets for disease treatment [212]. Despite these advances, the application of iPSC-induced vascular organoids in MMD remains underexplored. While stem cell therapy offers considerable promise, several challenges must be addressed, including safety concerns, variability in functional outcomes, and the limited physiological relevance of current preclinical models. Advances in multi-lineage organoid systems, humanized models, and standardized differentiation protocols may help overcome these limitations, providing more robust platforms for elucidating disease mechanisms and accelerating the development of effective therapies for MMD. However, stem cell therapy remains inherently complex due to challenges in identifying optimal cell sources, developing efficient delivery systems, and understanding underlying molecular mechanisms and regulatory pathways. Further research is urgently needed to address these barriers and enable its clinical translation.

#### iPSC-based Cell Therapy Combined with CRISPR

Gene editing technologies enable precise modification of genetic sequences and have become indispensable tools in biological research and therapeutic development. Among all the gene editing methods (TALEN, Cre-loxP, et al.), CRISPR/Cas9 gene editing holds great therapeutic potential for treating diseases. Recently, Feng Zhang discovery a new gene editing system called TIGR-Tas system, which exhibited greater targeting flexibility and less delivery difficulty [213]. For example, in CAR-T cell immunotherapy for tumor treatment, the knockdown of genes such as Pdcd1, TRAC, and TRBC using CRISPR/Cas9 can improve therapeutic efficacy while reducing side effects like cytokine release syndrome and improving the cytotoxicity of CAR-T cells [214]. Through CRISPR-mediated homology-directed repair (HDR), targeted gene repair or knockout of disease-causing mutations can be achieved. For instance, in the treatment of β-thalassemia and sickle cell anemia (an inherited disease caused by single base mutation in the globin gene), CRISPR-mediated knockout of BCL11A effectively upregulates fetal hemoglobin (HbF) expression, and then concentrated the repaired hemopoietic stem cells (HSCs) that carried the corrected cell genes injected into mice disease model. The therapy with CRISPR/cas9 successfully compensated the defective hemoglobin and ameliorated disease symptoms [215]. what’s more, the following study found that gRNA-68 CRISPR/Cas9 targeting HBG1 and HBG2 promoters of CD34^+^ hematopoietic progenitors stem cells produced no off-target mutations and produced high levels of fetal hemoglobin after differentiation in vitro or xenotransplantation into immunodeficient mice, which shows safety and efficacy in treatment with sickle cell anemia [216].

The combination of CRISPR/Cas9 with iPSCs offers a powerful strategy for treating genetic disorders. Disease-causing mutations can be corrected in patient-derived iPSCs, which are then differentiated into relevant cell types and transplanted autologously to restore tissue function, thereby avoiding immune rejection and ethical concerns. Numerous iPSC-based therapies are currently in clinical trials, including retinal pigment epithelial cells for age-related macular degeneration(AMD) [217], dopaminergic neurons for Parkinson’s disease [218], cardiomyocytes for myocardial infarction [219], cholangiocytes for hereditary hepatobiliary disorders [220], brain cells for psychiatric disorders [221], and hepatocytes for liver cancer [222]. For example, Romano et al*.* used CRISPR-Cas9 to generate PKD1-knockout iPSC lines that retained pluripotency and differentiation capacity, providing a valuable model for studying autosomal dominant polycystic kidney disease (ADPKD) and drug screening [223]. Similarly, Kang et al*.* achieved homozygous CCR5 knockout in iPSCs, which retained pluripotent characteristics and differentiated into hematopoietic cells and macrophages resistant to CCR5-tropic HIV infection, highlighting the promise of this combined strategy for HIV therapy [224]. Furthermore, CRISPR technology enables precise correction of pathogenic genetic mutations responsible for movement disorders, while iPSCs serve as an ideal autologous cell source capable of differentiating into functional neurons for transplantation therapy. In this approach, patient-derived iPSCs undergo CRISPR/Cas9-mediated genetic correction, followed by differentiation into specific subtypes, such as dopaminergic neurons. These genetically corrected neurons can then be transplanted into degenerating brain regions to restore motor function. For instance, in a Parkinson’s disease (PD) model, transplantation of dopaminergic neurons derived from CRISPR-corrected iPSCs effectively replenishes dopamine neurotransmitter levels, thereby alleviating motor deficits and improving neurological outcomes [225].

This combined approach leverages precise genome editing and iPSC-based regenerative therapy. Importantly, to achieve in vivo gene editing, effective delivery of CRISPR/Cas9 components (such as Cas9 protein and sgRNA) to target cells or tissues is essential. Both viral and non-viral vectors can be used for delivery. Adeno-associated virus (AAV) has emerged as a leading delivery vehicle due to its low pathogenicity, minimal immunogenicity, sustained expression, and broad tropism [226, 227]. Studies in mice have confirmed that AAV and lipid nanoparticles (LNP) can mediate precise genetic corrections in hepatocytes without detectable off-target effects [228].AAV has been successfully used to deliver homologous recombination templates alongside CRISPR-Cas9 to correct point mutations in human retinal organoids derived from iPSCs [229]. Zhang et al*.* packaged truncated saCas9 and sgRNA into a single AAV vector for in vivo genome editing in a DMD mouse mode [230], while Bengtsson et al. demonstrated HDR-mediated editing of DMD-related genes in muscle tissue using AAV-delivered CRISPR/Cas9 [231]. Alessio D. Nahmad et al. engineered a dual-AAV system to deliver SaCas9-sgRNA and an HIV-bNAb-3BNC117 repair template, enabling in vivo editing of B cell immunoglobulin genes to produce neutralizing antibodies against HIV, illustrating the potential for one-time genetic immunotherapy [232].

Apart from the CRISPR/Cas9 together with homology-directed repair to correct single base mutation, David Liu developed base editing system, with which can covert cytidine to thymine (CBE) or adenine to cytidine (ABE) [233].Base editing is a new type of gene editing technology that can directly modify a single base without nducing double-strand breaks, offering a safer alternative for correcting point mutations. Base editing can reduce the DNA damage and improve the efficiency of gene editing. Liang et al*.* first applied base editing to correct an HBB mutation in human embryos [234], and subsequent studies showed that ABE can repair HBB mutations in hematopoietic stem cells and restore β-globin expression [235].

**Fig. 4:**
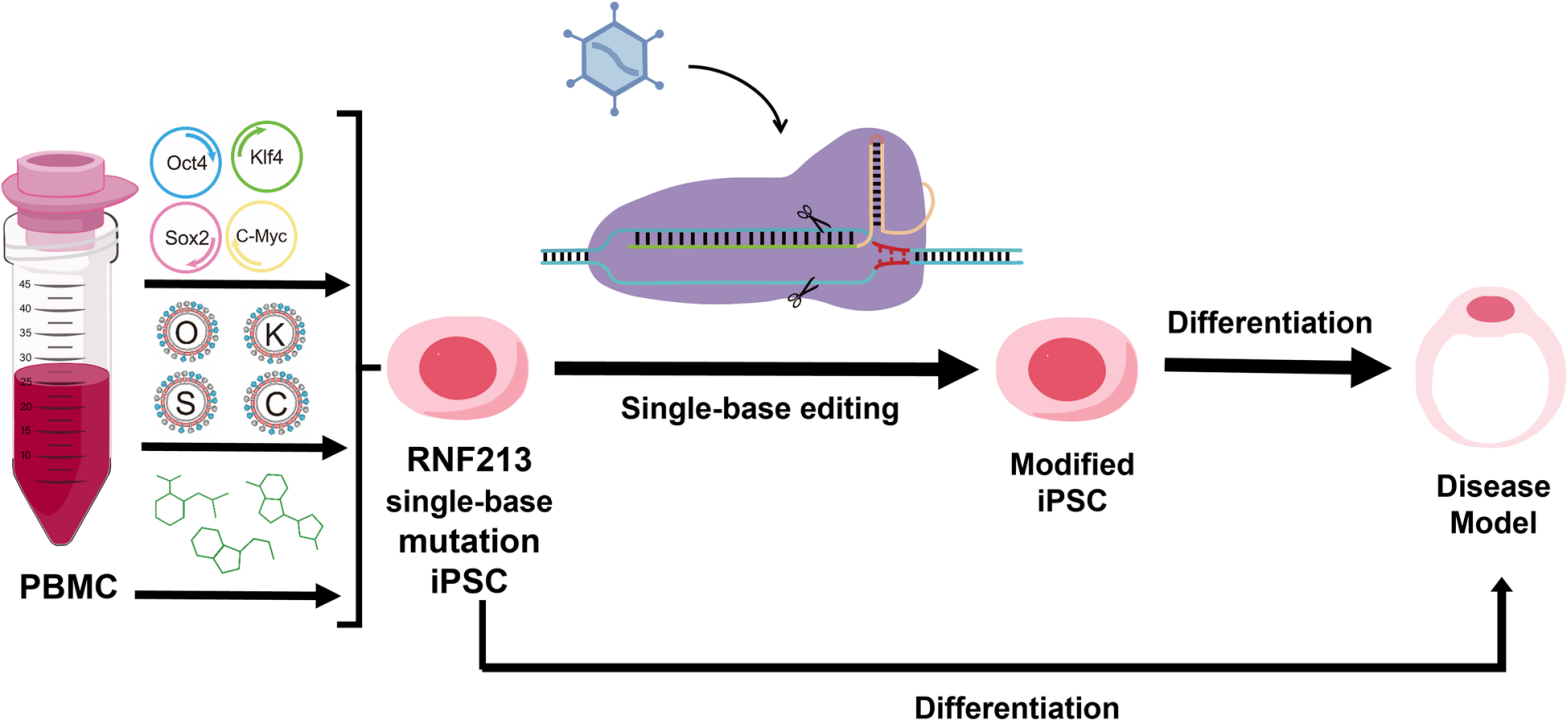
Emerging therapeutic strategies: Cell-based and gene-targeting interventions for Moyamoya disease. This schematic illustrates the experimental workflow for creating a patient derived vascular organoid model of Moyamoya disease using somatic reprogramming and gene editing technology

To investigate the pathogenic mechanisms of genetic mutations in MMD (e.g., RNF213), we propose an integrated strategy combining patient-derived iPSCs, CRISPR/Cas9-mediated Genetic correction, and 3D vascular organoid differentiation (Fig. 4). This approach enables the generation of isogenic controls and disease models that recapitulate key pathological features including vascular stenosis and aberrant collateral formation while preserving patient genetic backgrounds. Through multi-omics profiling, functional analyses of cell–cell interactions, and signaling pathway studies, this approach provides a physiologically relevant system for elucidating genotype–phenotype correlations, identifying novel therapeutic targets, developing personalized treatments, and deciphering disease mechanisms by merging advanced genome editing, organoid technology, and high-throughput screening.

#### Targeting Inflammation and Vascular Remodeling: Emerging Therapeutic Strategies

As previously noted, MMD vasculopathy is associated with chronic inflammation. For example, the MD2/TLR4 pathway recognizes damage-associated molecular patterns (DAMPs) such as oxidized low-density lipoprotein (oxLDL), leading to NF-κB activation and subsequent endothelial inflammation and stenosis [236]. Accordingly, small-molecule inhibitors (e.g., Resatorvid) or monoclonal antibodies targeting MD2/TLR4 may serve as adjunct anti-inflammatory therapies, particularly in advanced disease stages [237].

Therapeutic strategies may also focus on modulating the RNF213 pathway. For example, in cells with RNF213 mutations (especially p.R4810K), Cav-1 ubiquitination is significantly weakened, leading to its abnormal aggregation on the cell membrane, thus affecting caveolae structural stability and endothelial function. This mechanism offers a rationale for drug screening aimed at modulating Cav-1 degradation or enhancing RNF213 activity [238]. Additionally, DLL4-Notch1 antagonists such as DAPT have been shown to promote functional collateral circulation in murine models [239].Abnormal pericyte proliferation via the PDGFRβ pathway has also been implicated in aberrant angiogenesis and vascular remodeling [240]. Imatinib, a tyrosine kinase inhibitor targeting PDGFRβ, induces apoptosis and suppress proliferation of PDGFRβ^+^ pericytes, thereby reducing microvessel density and compromising vascular stability [241]. In experimental models of extracerebral spinal cord injury (SCI), imatinib-mediated inhibition of the PDGF-BB/PDGFRβ signaling pathway effectively attenuated fibrotic scar formation, promoted axonal regeneration, and reduced inflammation [242].Based on these findings, we hypothesize that PDGFRβ inhibitors such as imatinib may represent a promising therapeutic strategy for mitigating vascular stenosis in MMD, potentially opening new avenues for targeted medical therapy.

Although multiple molecular pathways have been identified as potential therapeutic targets, their clinical applicability hinges on validation through rigorous preclinical and clinical studies. The emerging therapeutic candidates and ongoing clinical evaluations represent a particularly promising area of development, which we will explore in the following section.

#### Preclinical and Clinical Studies of Emerging Therapeutic Strategies

Recent clinical research in MMD has broadened the therapeutic focus beyond surgical revascularization to include vascular protection, collateral promotion, and immunomodulation. In the realm of antiplatelet and vascular-active therapies, nationwide and multicenter studies in South Korea have demonstrated that cilostazol—a phosphodiesterase-3 inhibitor—is associated with improved long-term survival compared to other antiplatelet agents, alongside benefits in cerebral perfusion and cognitive function [243]. Follow-up imaging studies further revealed favorable vascular remodeling in patients receiving cilostazol [172].

In the area of remote ischemic conditioning (RIC), which aims to enhance endothelial-immune homeostasis, RIC has progressed to randomized controlled trials evaluating its efficacy in improving postoperative recovery in adult MMD patients [244]. Preliminary pilot investigations have already confirmed its safety and suggested promising effects on neurological outcomes [245]. Concerning perioperative complications, particularly hyperperfusion syndrome mediated by MMP-9-driven inflammation, retrospective controlled analyses indicate that minocycline may attenuate postoperative neurological deterioration [246]. Simultaneously, regenerative medicine approaches are gaining traction. A first-in-human phase I trial is currently assessing the safety and feasibility of combining iPSC-derived exosomes with temporal muscle flap transplantation during revascularization surgery [247]. Complementary translational studies profiling serum and CSF exosomes from MMD patients provide mechanistic insights and candidate biomarkers to support these innovative strategies [248, 249].

Collectively, these efforts reflect a shift toward mechanism-driven therapeutic innovation in MMD. However, the field still awaits larger, randomized controlled trials to confirm efficacy and translate preclinical promise into clinical practice. A summary of relevant treatment strategies is provided in Table 4.

**Table 4:** Emerging Therapeutic Strategies and Clinical Trials in Moyamoya Disease

Pathway/Target	Strategy	Mechanism of Action	Preclinical Evidence	Clinical Evidence
**TLR4/MD2–** **NF-κB** [236]	Resatorvid (TAK-242), anti-TLR4 mAb	Suppresses endothelial inflammation and vascular stenosis	Stroke/vascular injury animal models	No MMD-specific trial; TAK-242 tested in sepsis (Phase II)
**RNF213–Cav-1** [238]	Cav-1 degradation modulators	Restores caveolae stability, improves EC function	In vitro RNF213 mutant cells	None
**DLL4–Notch1** [239]	DAPT (γ-secretase inhibitor)	Promotes collateral circulation formation	Mouse ischemia models	None
**PDGFRβ** [240]	Imatinib	Inhibits pericyte proliferation, reduces fibrosis and stenosis	SCI and vascular remodeling models [242]	Approved for CML; no MMD-specific trial
**iPSC therapy** [98]	Patient-derived vascular cells/organoids	Restores vascular/endothelial function	iPSC-based MMD models	None
**iPSC + CRISPR**	Gene-corrected iPSCs (RNF213, others)	Personalized regenerative therapy	Conceptual framework; early rare disease models	None
**MSC/NSC therapy** [207]	Allogeneic/autologous transplantation	Neuroprotection and vascular repair	Stroke models	Some Phase I/II in stroke, not MMD

## Conclusion and Future Perspectives

### Genetic and Clinical Landscape of Moyamoya Disease

While MMD demonstrates a striking ethnic disparity, with highest prevalence in East Asia, its increasing recognition across diverse populations underscores the interplay of genetic susceptibility with environmental and immunological factors. Significant advances have been made in uncovering the genetic architecture of MMD, most notably through the identification of RNF213 as a major susceptibility gene, particularly in East Asian cohorts. Nevertheless, the relatively low penetrance of even high-risk alleles such as p.R4810K suggests that additional genetic modifiers, epigenetic regulation, and exogenous triggers are integral to disease manifestation. Clinically, the diagnosis of MMD relies on a combination of characteristic angiographic findings and the exclusion of alternative vasculopathies. Digital subtraction angiography remains the diagnostic gold standard, though advances in high-resolution magnetic resonance angiography and hemodynamic imaging have enhanced non-invasive monitoring and preoperative planning. Current management is predominantly surgical, with direct and indirect revascularization strategies aimed at restoring cerebral perfusion. However, these interventions do not reverse the underlying pathobiology, highlighting the urgent need for medical therapies that target disease mechanisms.

The molecular pathogenesis of MMD involves dysregulated angiogenesis, aberrant epigenetic regulation, chronic inflammation and environmental triggers, driven by interactions between endothelial dysfunction, smooth muscle cell proliferation and immune activation. Recent multi-omics approaches have revealed disruptions in metabolic pathways, RNA splicing, and extracellular matrix organization, offering new insights into disease mechanisms and potential biomarkers.

Looking forward, the integration of patient-derived iPSCs with Gene editing and 3D vascular organoid technologies provides a transformative platform for elucidating genotype–phenotype relationships, modeling patient-specific vasculopathy, and screening novel therapeutics.

### Breaking Through Bottlenecks in Moyamoya Disease Research

Currently, the study of MMD facing mainly two bottlenecks: one is that the tissue samples of intracranial arteries and smog like vessels from patients are difficult to obtain due to sample scarcity and ethical restriction. Previous studies using samples from patients' peripheral blood could not accurately reveal the Gene expression differences and interaction networks of different cell subsets in the disease microenvironment, such as which endothelial cell subsets drive vascular stenosis and which immune cells participate in inflammation. The other is the existing models are hindered by interspecies disparities that prevent the formation of characteristic collateral vessels in animals, and by the structural oversimplification inherent in 2D cell culture systems. So far, research into the pathogenesis of MMD remains at a nascent stage. Current treatment is largely restricted to surgical revascularization, which can improve cerebral perfusion but carries risks of craniotomy-related sequelae such as cognitive impairment or memory deficits. Moreover, surgery is unsuitable for early intervention and does not target the underlying molecular drivers of disease progression. There is therefore an urgent need to elucidate the cellular and molecular mechanisms of MMD from a multidimensional perspective, which would provide a scientific foundation for novel therapeutic strategies and expand rehabilitation options for this rare disease.

Advances in somatic reprogramming and cell therapy technologies offer promising avenues for progress. The combination of CRISPR/Cas9-mediated gene editing and adenovirus-assisted homologous recombination in patient-derived iPSCs enables precise genetic correction of RNF213 mutations and other disease-associated variants. This gene-editing strategy, coupled with stem cell-based approaches, opens possibilities for non-invasive early intervention without requiring craniotomy. Although challenges remain, such as incomplete reprogramming, potential of tumorigenesis and immune compatibility concerns [250–252], the iPSC-induced somatic cells or organoids have already demonstrated significant success in recapitulating key disease phenotypes. These human-relevant models provide unprecedented opportunities to investigate disease mechanisms in a controlled, patient-specific context, accelerating the development of targeted therapies for MMD.

### Integrative Insights into Moyamoya Disease

MMD represents a paradigmatic challenge in cerebrovascular research, characterized by elusive etiology, complex vascular remodeling, and limited therapeutic options. Current progress in epidemiological investigations, genetic studies—particularly concerning RNF213—and clinical management has greatly advanced our understanding of this rare disorder. In this review, we have synthesized current knowledge on the typical phenotypic manifestations, population-based incidence patterns, susceptibility genes, potential molecular mechanisms, and emerging iPSC-based therapies combined with CRISPR gene editing. Over recent years, insights have accumulated regarding the genetic susceptibility and polymorphism of RNF213, as well as immune cell alterations during MMD progression. However, the pathogenesis of MMD involves multifactorial interactions, including genetic predisposition (RNF213), infections, epigenetic modifications, and inflammation mediated by immune dysregulation. Moreover, it remains to be explored that which cell-type-specific mechanisms (such as endothelial cell dysfunction and immune microenvironment disorder) lead to vascular remodeling. In addition, the key biological events such as endothelium-smooth muscle cell interaction and extracellular matrix remodeling during the formation of abnormal vascular network have not been systematically studied.

In summary, future research should prioritize the development of physiologically relevant models—particularly patient-derived iPSC-based vascular organoids to accurately capture human-specific disease mechanisms. Concurrent efforts should focus on dissecting cell-type-specific pathological interactions and advancing gene-editing and stem cell-based strategies toward preclinical validation. Integrating these scientific and translational approaches will be essential to transforming mechanistic insights into effective therapies and improving clinical outcomes for MMD patients.

## Data Availability

Not applicable.
